# Integrating molecular characterization and metabolites profile revealed *Ct*CHI1’s significant role in *Carthamus tinctorius* L.

**DOI:** 10.1186/s12870-019-1962-0

**Published:** 2019-08-27

**Authors:** Dandan Guo, Yue Gao, Fei Liu, Beixuan He, Xinlei Jia, Fanwang Meng, Hai Zhang, Meili Guo

**Affiliations:** 1 Pharmacy college, Second Military Medical University, Shanghai, China; 2 Department of Chemistry, Shanghai University, Shanghai, China; 3 Department of Pharmacy, Shanghai First Maternity and Infant Hospital, Tongji University School of Medicine, Shanghai, China

**Keywords:** *Carthamus tinctorius* L., Model plant, Flavonoid biosynthesis, Chalcone isomerase, Metabolic database

## Abstract

**Background:**

As a traditional Chinese herb, safflower (*Carthamus tinctorius* L.) is valued for its florets to prevent cardiovascular and cerebrovascular diseases. Basing on previous chemical analysis, the main active compounds are flavonoids in its florets. Although flavonoid biosynthetic pathway has been well-documented in many model species, unique biosynthetic pathway remains to be explored in safflower. Of note, as an important class of transitional enzymes, chalcone isomerase (CHI) has not been characterized in safflower.

**Results:**

According to our previous research, CHIs were identified in a safflower transcriptome library built by our lab. To characterize CHI in safflower, a CHI gene named *Ct*CHI1 was identified. A multiple sequences alignment and phylogenetic tree demonstrate that *Ct*CHI1 shares 92% amino acid identity and close relationship with CHI to *Saussurea medusa*. Additionally, subcellular localization analysis indicated *Ct*CHI1-GFP fusion protein was mainly in the cell nucleus. Further, we purified *Ct*CHI1 protein from *E. coli* which can effectively catalyze isomerization of 2′,4′,4,6′-tetrahydroxychalcone into naringenin in vitro. Via genetic engineer technology, we successfully obtained transgenic tobacco and safflower lines. In transgenic tobacco, overexpression of *Ct*CHI1 significantly inhibited main secondary metabolites accumulation, including quercetin (~ 79.63% for ovx-5 line) and anthocyanins (~ 64.55% for ovx-15 line). As shown in transgenic safflower, overexpression of *Ct*CHI1 resulted in upstream genes *Ct*PAL3 and *Ct*C4H1 increasing dramatically (up to ~ 3.9fold) while *Ct*4CL3, *Ct*F3H and *Ct*DFR2 were inhibited. Also, comparing the whole metabolomics database by PCA and PLS-DA between transgenic and control group, 788 potential differential metabolites were marked and most of them displayed up-regulated trends. In parallel, some isolated secondary metabolites, such as hydroxysafflor yellow A (HSYA), rutin, kaempferol-3-*O*-β-rutinoside and dihydrokaempferol, accumulated in transgenic safflower plants.

**Conclusions:**

In this study, we found that *Ct*CHI1 is an active, functional, catalytic protein. Moreover, *Ct*CHI1 can negatively and competitively regulate anthocyanins and quercetin pathway branches in tobacco. By contrast, *Ct*CHI1 can positively regulate flavonol and chalcone metabolic flow in safflower. This research provides some clues to understand CHI’s differential biochemical functional characterization involving in flavonoid pathway. More molecular mechanisms of CHI remain to be explored in the near future.

**Electronic supplementary material:**

The online version of this article (10.1186/s12870-019-1962-0) contains supplementary material, which is available to authorized users.

## Background

**Fig. 1:**
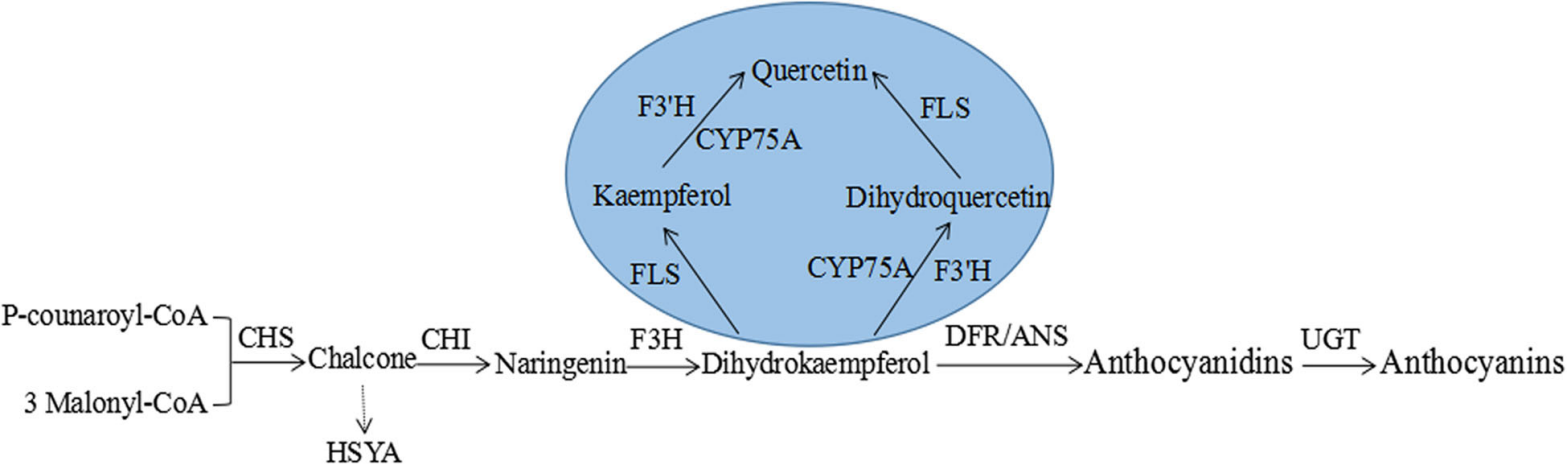
A schematic diagram of flavonoid pathway in plant. Notes: HSYA is a unique chalcone flavonoid compound in safflower. Dotted line arrow represent predicted route of HSYA biosythesis from safflower

Flavonoids represent a class of plant-specific secondary metabolites that have various functions during plant growth and development, including flower pigmentation, protection against UV irradiation, defending against the pathogen, pollen development, cell cycle regulation, and auxin transport [1–3]. In flavonoid biosynthetic pathway, the chalcone synthase (CHS) first produces chalcones, and then chalcone isomerase (CHI, EC 5.5.1.6) catalyzes the stereospecific conversion of chalcones into their corresponding (2*S*)-flavanones which initiate the flavonoid metabolic flow. Subsequently, the flavanone 3-hydroxylase(F3H) catalyzes the hydroxylation and conversion of flavanones into dihydroflavonols. Two consecutively acting enzymes, dihydroflavonol 4-reductase (DFR) and anthocyanidin synthase (ANS), boost the synthesis of anthocyanidins treating dihydroflavonols as substrate. The following glycosylation of anthocyanidins is generally mediated by UDP-glucose (Fig. 1). Many documentaries reported that although chalcone compounds can be isomerized non-enzymatically or non-chemically into (2*RS*)-flavanones in neutral solution, only (2*S*)-flavanones are significant intermediates for the subsequent flavonoid-like natural secondary products. For example, 2′, 4′, 4, 6′-tetrahydroxychalcone (chalcone naringenin) can rapidly isomerize into5, 7, 4′-trihydroxyflavanone (naringenin), while the isomerization reaction of 6′-deoxychalcone into 5-deoxyflavanone is slower owing to the difference of substrate intramolecular hydrogen bond [4].

The cDNA cross-hybridization and antigenic cross-reactivity indicated that the various substrate specificities of CHIs was caused by the CHI proteins structures between leguminous and nonleguminous plants [5]. CHIs are classified into 4 subfamilies (type I to type IV) [6], and their distributions are highly family-specific [4, 7]. Type I CHIs, generally available in nonlegumes, can easily isomerize only 6′-hydroxychalcone to produce (2*S*)-naringenin, whereas type II CHIs, specific in leguminous plants, can convert 6′-hydroxychalcone and 6′-deoxychalcone to (2*S*)-naringenin and (2*S*)-liquiritigenin, respectively. Type III CHIs are widely present in green algae and land plants, while type IV CHIs are restricted to land plants. Structural analysis showed that all CHIs share a similar backbone conformation [8, 9]. However, type III and type IV CHIs don’t possess CHI activity, which led to the renaming of both types of CHIs as CHI-like proteins (CHIL). Type III CHI folds play a significant role in fatty acid metabolism in plant [8]. Interestingly, biochemical evidence suggested that leguminous plants such as *Glycyrrhiza echinate* [4] includes type I and type II CHI. CHI has often been reported to be unique plant-specific maker gene [5]. However, via chalcone isomerase family sequences and three-dimensional folds analysis, bacterial and fungal species were demonstrated to have chalcone isomerase-like genes and incline to lack the orthologs of chalcone synthase [10]. The molecular weight of type I and type IICHIs is about 24 ~ 29kDalton. Genes that encode both types of CHIs have been cloned and characterized from different plant species [11–16]. Based on the deduced amino acid sequences alignment, the same type of CHI showed about ~ 70% identity, whereas type I and II CHIs is only around 50% [7]. The structure of type I and type II CHI has been also solved out by X-ray crystallography. In *Arabidopsis thaliana,* type I CHI-fold family have been dissected*,* including ligand-binding properties, crystal structures and in vivo functional characterization [8]. Among that, the structure of the alfalfa (*Medicago sativa*) CHI (type II CHI) protein provided insight into details of the dynamic reaction mechanism [17].

Our previous studies have analyzed a normalized cDNA library and gene chip data of safflower systematically [18]. All 23 flavonoid-related genes were listed and quantitatively analyzed by chip and qPCR. Among these, only two CHIs were marked based on functional annotation while one CHI also acts as flavonoid-enhancer and the other one is pure chalcone isomerase. In this article, we will describe the cloning and biochemical characterization of a true CHI gene from safflower. Furthermore, we also explored the potential role of CHI in secondary metabolite biosynthetic pathway.

## Results

### Isolation and characterization of *Ct*CHI1

**Fig. 2:**
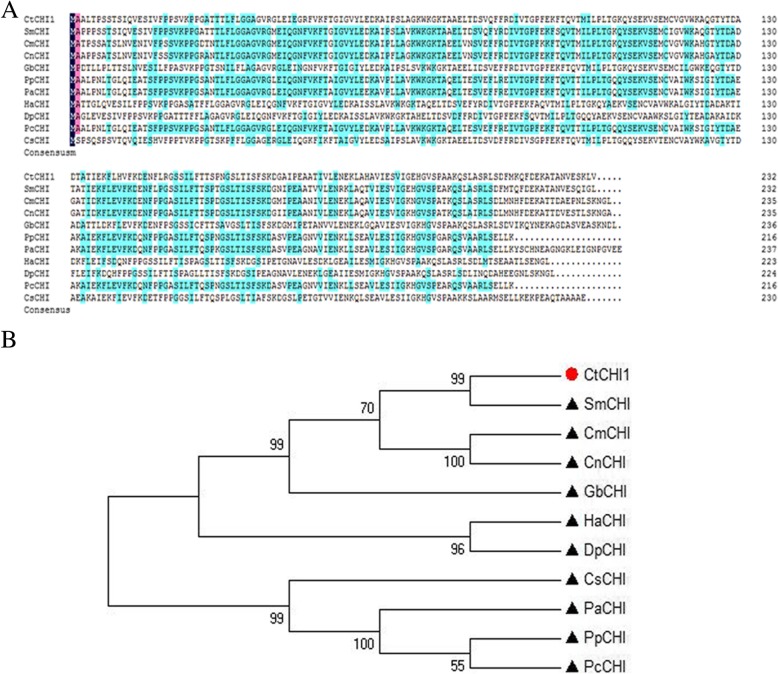
**a** Alignment of deduced *Ct*CHI1 amino acid sequences with other plant species. Identical residues are highlighted on a black background, and similar residues are highlighted on a pink and blue background. The GenBank accession numbers are as follows: *Sm*CHI: Q8LKP9.1 from *Saussurea medusa*; *Cm*CHI: A1E261.1 from *Chrysanthemum x morifolium*; *Cn*CHI1: ASX95441.1 from *Chamaemelum nobile*; *Gb*CHI: BAJ17665.1 from *Gynura bicolor*; *Pp*CHI: XP_007218371.1 from *Prunus persica*; *Pa*CHI: AJO67964.1 from *Prunus avium*; *Ha*CHI: XP_022013593.1 from *Helianthus annuus*; *Dp*CHI: BAJ21533.1 from *Dahlia pinnata*; *Pc*CHI: AKV89240.1 from *Prunus cerasifera*; *Cs*CHI: ASU87415.1 from *Camellia sinensis*. **b** Unrooted phylogram comparison of the amino acid sequences of *Ct*CHI1 with other functionally characterized CHI proteins. The sequences used are the same as in Fig. 2a. The phylogenetic tree was constructed by MEGA, after alignment using ClustalX software. Node support was estimated using neighbor-joining bootstrap analysis (1,000 bootstrap replicates)

A full-length cDNA sequence of *Ct*CHI1 gene was isolated from *Carthamus tinctorius*L. and named *Ct*CHI1 (GenBank accession no. MF421811). This gene encodes a predicted polypeptide of 232 amino acids. The deduced amino acid sequence of the cDNA showed that it encoded a polypeptide of approximately 24.9 kDa and an isoelectric point of 5.8. A multiple-sequence alignment by DNAMAN demonstrated that CHIs have 92 and 85% amino acid identity to *Saussurea medusa* CHI (GenBank accession No.Q8LKP9.1) and *Cynara cardunculus var. scolymus* CHI (KVI06946.1), respectively (Fig. 2a). A phylogenetic tree (Fig. 2b) generated by the neighbor-joining (NJ) method based on the putative amino acid sequences indicated that *Ct*CHI1 has close relationship with CHI from *S. medusa*, which can enhance apigenin biosynthesis by overexpression of the chalcone isomerase gene in hairy root cultures.

### Subcellular localization

**Fig. 3:**
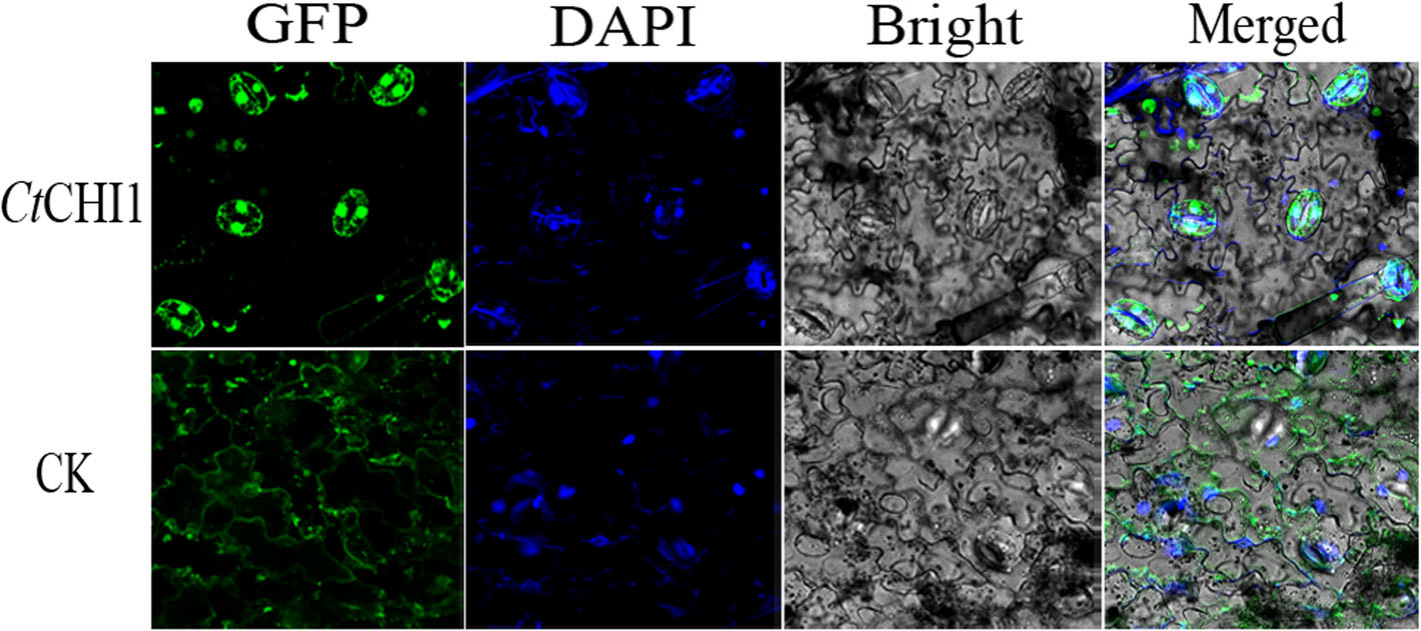
Subcellular localization of the *Ct*CHI1-GFP fusion protein. **a** Subcellular localization of the *Ct*CHI1-GFP fusion protein in onion epidermal cells. GFP fluorescence (GFP; green pseudocolor), optical photomicrographs (bright field), DAPI and an overlay of bright and GFP fluorescence illumination and DAPI (merged) are shown; the arrows point to the nucleus of the cells. Data shown are representative of three independent experiments (n = 3)

To examine the localization of the *Ct*CHI1 protein, the *Ct*CHI1-GFP fusion construct, driven by the *Ca*MV 35S promoter, was transformed into *Agrobacterium tumefaciens* GV3101 and subsequently gently introduced into tobacco leaf cells by injection. The tobacco leaves were infiltrated on the downside of leaves with a 1 ml syringe without needle, prior to that, a needle was used to make a tiny hole so that the suspension buffer with constructed plasmid can go into the leaves smoothly. The infiltration of *Nicotiana benthamiana* would be very well controlled. The tobacco plants were placed in the light for 72 h following 24 h of darkness prior to microscopy imaging. The results showed that GFP fluorescence was detected in the nucleus when capturing the GFP signal in transient expression of *Ct*CHI1 tobacco. From the merged images of GFP, DAPI, and bright field, the GFP signal can be seen in the cell nucleus clearly in the *Ct*CHI1 group when the control group was localized in the cytoplasm and membrane (Fig. 3).

### Molecular docking analysis

**Fig. 4:**
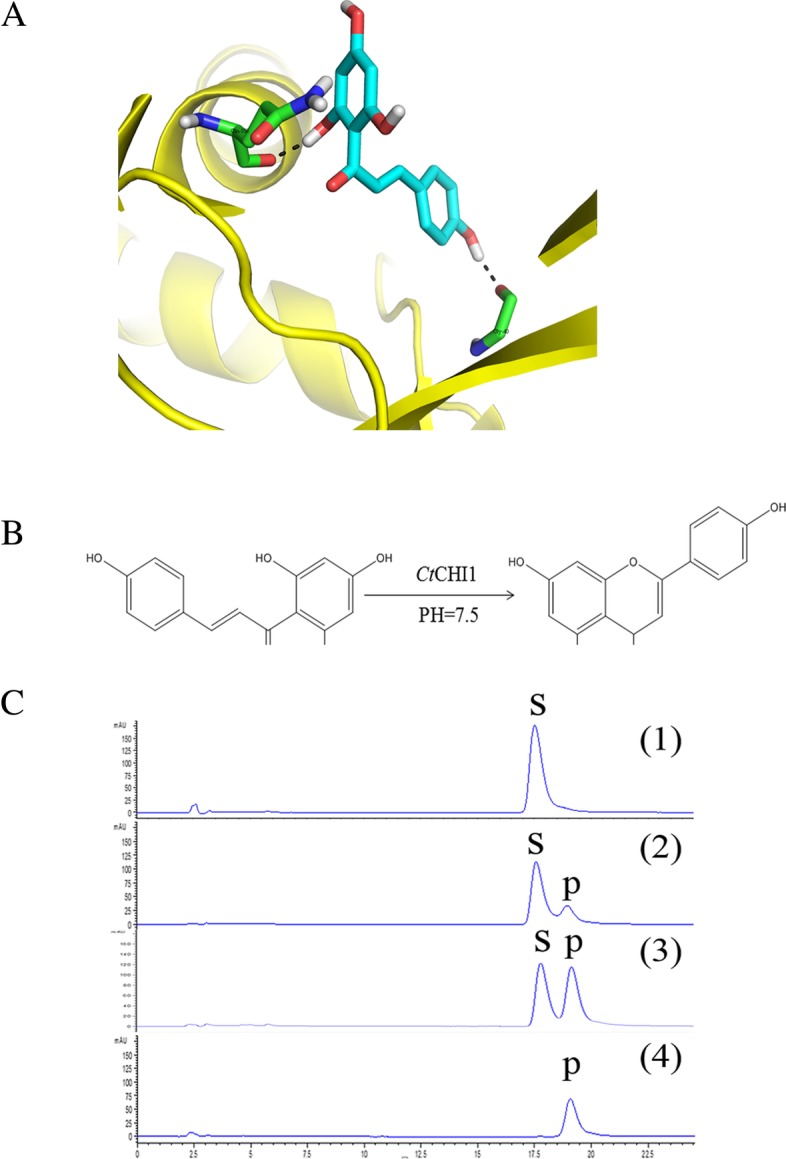
**a** Putative binding mode of 2′,4′,4,6′-tetrahydroxychalcone against the modelled protein structure. The protein is shown in cartoon and the 2′,4′,4,6′-tetrahydroxychalcone is in cryan. Residues forming hydrogen bonds have been labled (Gln108 and Gly40). **b** *Ct*CHI1 protein catalyzed reaction in *vitro*. Substrate (2′,4′,4,6′-tetrahydroxychalcone) and product (naringenin) compounds are drawn. **c** HPLC pattern displays reaction progress with enzyme and without enzyme. S: substrate compound, P: product compound. (1) substrate compound in reaction buffer (2) spontaneous catalytic reaction without enzyme (3) catalytic reaction with enzyme (4) product compound in reaction buffer

To reveal the potential role of *Ct*CHI1 in catalyzing the 2′,4′,4,6′ -tetrahydroxychalcone and understand the possible interaction modes [19], a molecular docking simulation was performed. The protein structure is built from its homology protein chalcone isomerase (PDB ID: 1EYQ) with Modeller (Fig. 4a). The structure proved to be reasonable by the Ramachandran plot (http://services.mbi.ucla.edu/SAVES/Ramachandran/) as well as by procheck [20, 21]. The results suggest that the enzyme is capable of binding with 2′,4′,4,6′ -tetrahydroxychalcone. The compound forms two hydrogen bonds with *Ct*CHI1 at the conversed catalytic pocket by Thr 105, Leu 104, Phe 50, Leu 41, Arg 39, Lys 112, Glu 111, and Lys 112.

### Functional expression of *Ct*CHI1 in *Escherichia coli*, purification, and enzymatic assay

The *Ct*CHI1 gene was heterologously expressed at 30 °C in *E. coli* BL21(DE3) pLyscells with an MBP-tag protein in the pMAL-c5x vector. The recombinant protein was obtained in a soluble fraction. Thus, the soluble fraction of recombinant protein was purified and concentrated by means of MBP’s affinity chromatography for maltose. Purified CHI migrates on sodium dodecyl sulfate–polyacrylamide gel electrophoresis (SDS-PAGE) gels with a molecular mass of 24.9 kDa. To verify the function of *Ct*CHI1 in vitro, we attempted to test a set of flavonoid compounds as substrates. In HEPES buffer, we figured out that the recombinant *Ct*CHI1 efficiently catalyzed the isomerization of 2′,4′,4,6′-tetrahydroxychalcone to produce naringenin at 30 °C (Fig. 4b and c), although 2′,4′,4,6′-tetrahydroxychalcone can also be isomerized to naringenin in a non-enzymatic manner [22]. CHI-catalyzed reaction of 2′,4′,4,6′-tetrahydroxychalcone was negligible and took place very slowly at 4 °C under the assay conditions employed in the present study. The optimum pH for the *Ct*CHI1-catalyzed isomerization of 2′,4′,4,6′-tetrahydroxychalcone is 7.5 [23]. The result demonstrated that *Ct*CHI1 belongs to type I CHIs.

To confirm the enzymatic kinetic value of *Ct*CHI1, the standard curves of naringenin and 2′,4′,4,6′-tetrahydroxychalcon (chalcone naringenin) were drew first, respectively (Additional file 1: Figure S1). Basing on the curves, we precisely detected substrate and product compounds amount in 180 min, and the data were shown in only 20 min (Additional file 1: Figure S1) (these curve data include spontaneous rate). It has been reported utilizing 2′,4′,4,6′-tetrahydroxychalcone as substrates, Kcat = 11,180 ± 1380 min^− 1^, Km = 112 ± 28 μm, over the spontaneous reaction rate with soybean CHI. Notably, kinetic analysis, in the presence of HEPES (pH = 7.5), indicated that *Ct*CHI1 efficiently catalyzed the conversion of chalcone naringenin into naringenin, with Km = 1. 585 mM and Kcat =1.87 × 10^9^ min^− 1^. The enzymatic activity of *Ct*CHI including spontaneous reaction is faster than CHI in *Antirrhinum majus* L. (THC: Kcat = 1.7 [s − 1] Km = 7.0 [μM], PHC: Kcat = 0.16 [s − 1] Km = 2.3 [μM]) [22] and also stronger than 3 CHIs in *Lotus japonicus* [7].

### Transcript expression patterns of *Ct*CHI1 in tobacco and safflower petal

**Fig.5:**
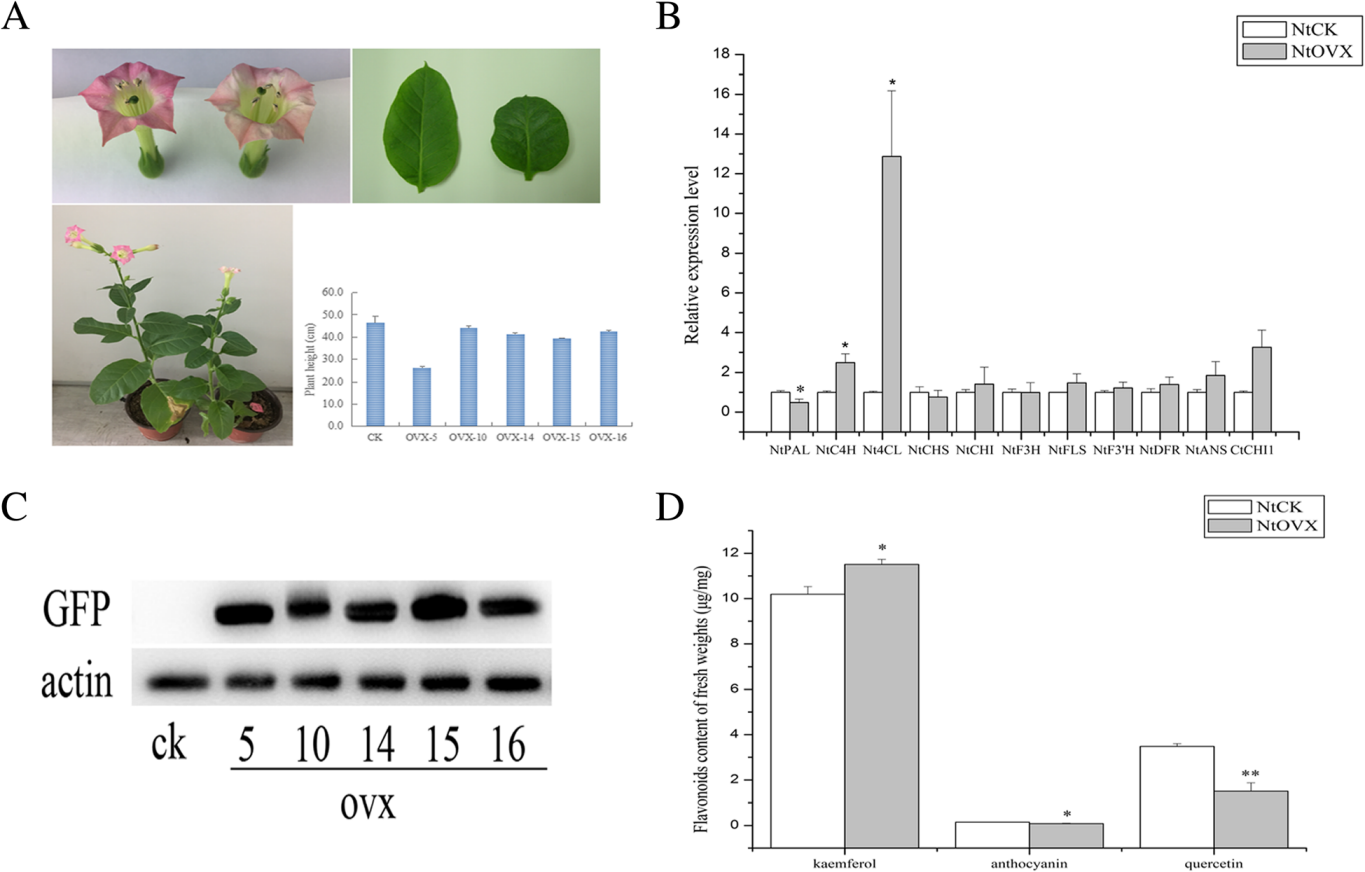
**a** Phenotype of *Ct*CHI1 over-expression in transgenic tobacco. Representative images are derived from empty-vector (on the left) and ovx-5 (on the right) lines. **b** Relative expression level of flavonoid-related genes in transgenic tobacco. **c** Western blot with anti-GFP antibody in transgenic tobacco lines. CK is as a negative control. **d** Flavonoids content of fresh weights (μg/mg) in transgenic tobacco comparing to CK group. Error bar is mean ± SE, data represent biological duplication. * *p*≤0.05, ** *p*≤0.01

To investigate the transcription expression level of related flavonoid genes in tobacco and safflower, quantitative real-time polymerase chain reaction (PCR) was performed with gene-specific primers. The expression patterns of *Ct*CHI1 and related flavonoid genes were examined by extracting total RNA from the petals. As shown in Fig. 5b, the relative transcription level of *Ct*CHI1 increased strongly in overexpression *Ct*CHI1 tobacco compared to the control group and had the highest expression level (~ 6.37-fold) observed in ovx-10 tobacco petals, whereas a higher level was found in ovx-14 (~ 3.17-fold) and ovx-16 (~ 3.48-fold) lines (Additional file 2: Figure S2A).

The quantitative PCR results indicated that upstream genes of the phenylpropanoid pathway, including *Nt*C4H and *Nt*4CL increased strongly, whereas *Nt*PAL decreased slightly in transgenic tobacco. In addition, the expression levels of flavonoid biosynthetic downstream genes were dramatically changed but not regularly, including *Nt*FLS, *Nt*F3′H, *Nt*DFR, and *Nt*ANS (Fig. 5b and Additional file 2: Figure S2A). This suggests that the down-regulated structural genes may own a potential competition directing to a different metabolic flux branch.

**Fig. 6:**
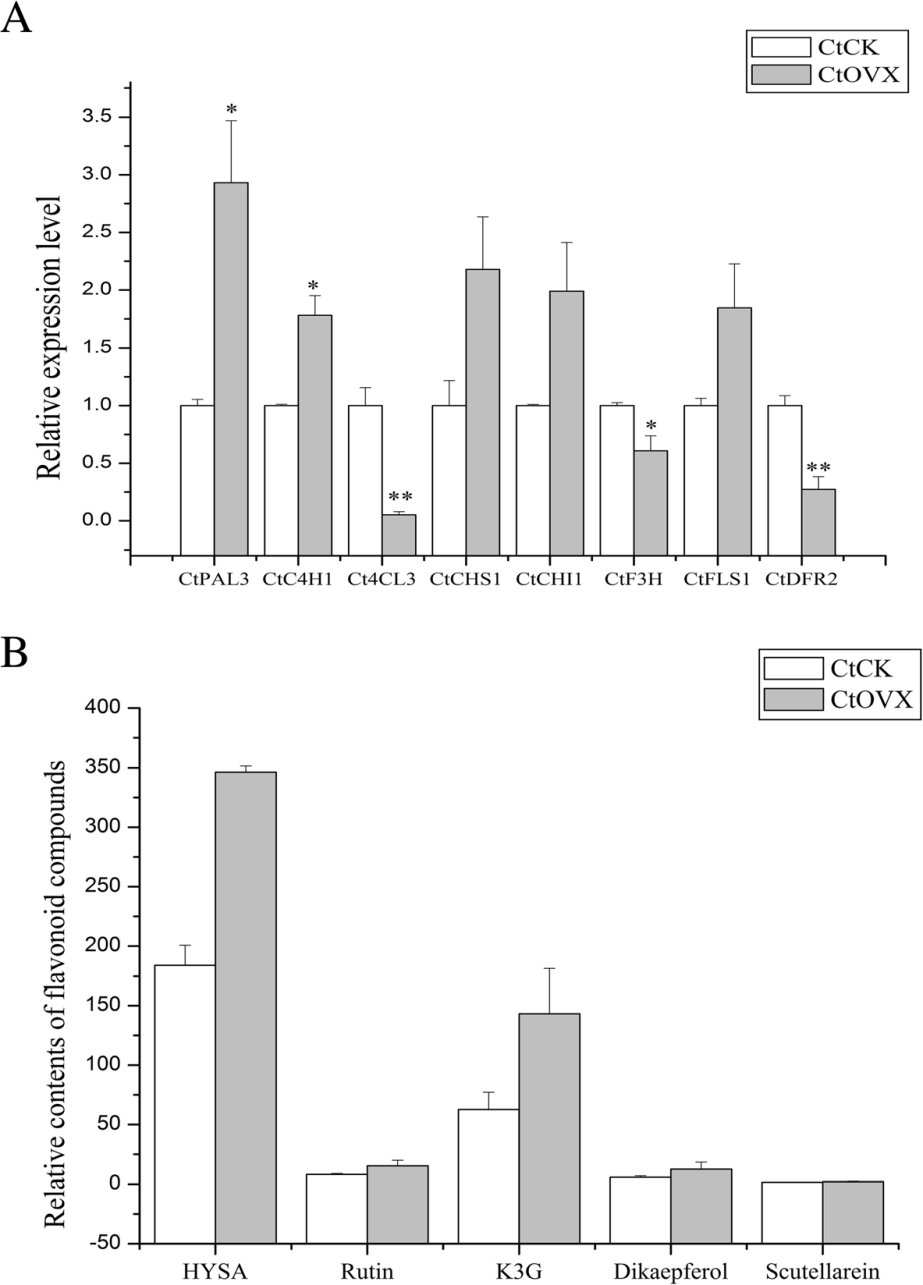
**a** Relative expression level of flavonoid-related genes in transgenic safflower. **b** Relative contents of flavonoid compounds implicating in flavonoids pathway. K3R: kaempferol-3-*O*-β-rutinoside; Dikaempferol: dihydrokaempferol. These selected genes are obtained from safflower transcriptome library. Error bar is mean ± SE, data represent biological duplication. * *p*≤0.05, ** *p*≤0.01

In parallel, for transgenic safflower, flavonoid-related genes expression levels are displayed in Fig. 6a, especially ovx-3, ovx-5, and ovx-6 (Additional file 3: Figure S3A). Apparently, endogenous *Ct*CHI1 was overexpressed up to 2.70fold in ovx-5 line. As shown in the figure, upstream genes *Ct*PAL3 and *Ct*C4H1 were up-regulated strongly appropriately 3.88- and 2.12-fold in ovx-3, whereas downstream genes *Ct*F3H and *Ct*DFR2 decreased obviously (≥50%) in ovx-3 and ovx-6 (Additional file 3: Figure S3A). This indicated overexpression of *Ct*CHI1 may restrain *Ct*DFR2 gene expression, which can potentially affect the anthocyanin pathway. In brief, the quantitative PCR results revealed that overexpression of *Ct*CHI1 has an individual and varied effect on flavonoid metabolite accumulation in tobacco and safflower.

### Western blotting

Using GFP-fusion protein antibody in Western blotting analysis, we found that the target protein displayed obvious differential expression level compared with negative control (CK). Actin acts as a reference protein. This result demonstrates that *Ct*CHI1 protein was overexpressed and protein expression levels are different in various transgenic tobacco lines (Fig. 5c).

### Flavonoid accumulation analysis in transgenic tobacco

Overexpression of the *Ct*CHI1 gene in tobacco was characterized. The phenotypic difference was clearly observed that the flower color of transgenic tobacco plants changed from pink to light pink (Fig. 5a). Moreover, the leaf became smaller, greener, and rounder in transgenic tobacco compared to empty vector control (Fig. 5a). However, by measuring the plant height, we figured out that the height was slightly shorter, in addition, the height of ovx-5 became shorter apparently without statistical difference (Fig. 5a). The high-performance liquid chromatography (HPLC) results (Fig. 5d) showed that anthocyanin and quercetin obviously decreased and kaempferol increased, in transgenic tobacco, which were corresponding to the color change and protein expression level changes. Kaempferol increased 11.84, 13.07, and 20.88% in ovx-5, ovx-14, and ovx-16 lines, respectively. By contrast, quercetin decreased the most robustly in ovx-5 line (~ 79.63%), and second most in ovx-15 line (~ 70.56%), whereas anthocyanins were suppressed significantly in each overexpression *Ct*CHI1 tobacco plant and the best line (ovx-15) increased by 64.55% (Additional file 2: Figure S2B). The results suggest that overexpression of *Ct*CHI1 in tobacco promoted kaempferol accumulation and inhibited anthocyanins and quercetin generation in flowers. In brief, overexpression of *Ct*CHI1 can cause the competition between kaempferol and quercetin in flavonol biosynthetic pathway and also negatively regulate the anthocyanin pathway branch in tobacco.

### Metabolomics analysis in transgenic safflower

**Fig. 7:**
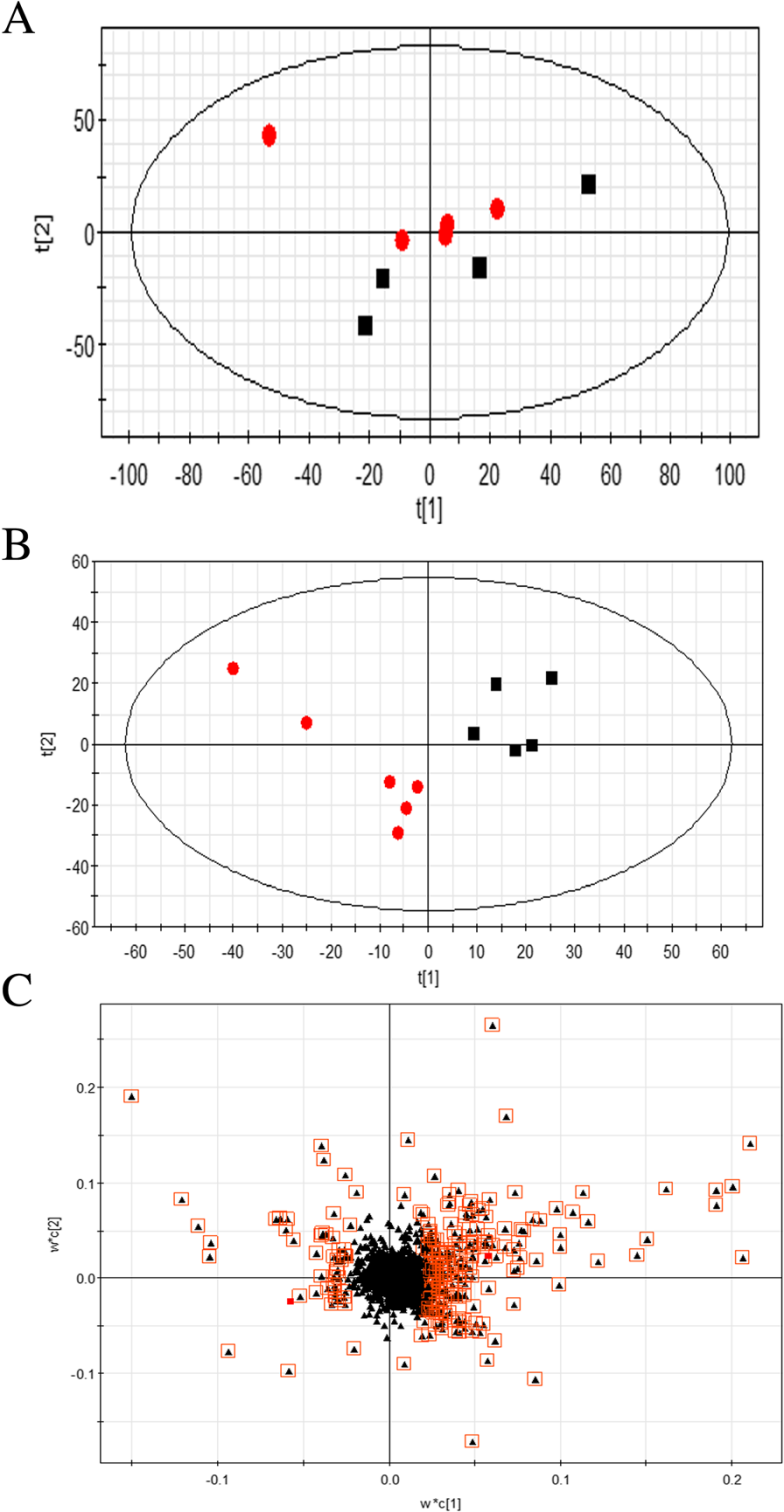
Metabolomic analysis in transgenic safflower compared to CK group. **a** PCA scores plot **b** PLS-DA scores plot **c** loading plot stemming from negative ion mode data sets of control group (filled black square) and ovx group (filled red circle)

To evaluate the whole metabolic database, the pattern recognition of PCA (principal component analysis) and PLS-DA (partial least squares discriminant analysis) was performed. The unsupervised PCA was utilized as an unbiased statistical method to investigate the general interrelation between groups and obtained a separation (Fig. 7a). This suggests a significant alteration in the metabolic profile induced by *Ct*CHI1 overexpression. In parallel, to discover the variation of metabolic profiles between the control and ovx groups, a supervised PLS-DA method was used to promote the metabolites detection, and the PLS-DA model from negative mode generated better differentiation ability between groups (Fig. 7b). The corresponding loading plots (Fig. 7c for negative ion mode) were used for selecting potential differential metabolites, in which the ions farthest away from the origin contribute significantly to the clustering of the two groups. Each spot represents an endogenous substance, and the spots located at the ends of the plot will contribute more to the differentiation between groups. Finally, according to the variable importance in the approach mentioned previously, a total of 788 differential metabolite ions from the negative ion mode were selected. The metabolites are summarized in Additional file 4: Table S1. Major differential metabolites remain to be identified by high-resolution mass spectrometry and MS/MS experiments.

Additionally, we detected some significant active compounds involved in the safflower flavonoid pathway, while standard compounds were stored in our laboratory. HSYA, rutin, kaempferol-3-*O*-rutinoside, and dihydrokaempferol increased strongly in some transgenic lines (Fig. 6b). Typically, for ovx-6 line, four secondary metabolites mentioned previously dramatically raised 3.11-, 3.04-, 3.50-and 4.27-fold, respectively (Additional file 3: Figure S3B).

## Discussion

CHI was a potential functional enzyme directing to produce various active flavonoid compounds. Despite extensive studies on CHI classification and cloning, little is known about the regulation of CHI on secondary metabolites in safflower [24–27]. In the present study, we carried out a sequence analysis of a pure *Ct*CHI1 gene and investigated its activity in vitro, as well as its transcript abundance and metabolic accumulation in both transgenic tobacco and safflower lines.

The ORF of *Ct*CHI1 was 1378 bp and encoded 232 amino acids. a multiple sequences alignment demonstrated that *Ct*CHI1shares 92% amino acid identity and close relationship with CHI from *S. medusa*. Generally speaking, chalcone isomerase catalyzes the intramolecular cyclization of 2′,4′,4,6′-tetrahydroxychalcone, derived from the upstream enzyme chalcone synthase, into (2S)-naringenin (5,7,4′-trihydroxyflavanone) under non-enzymic or enzymic condition [28, 29].In view of spontaneously cyclizing, CHI guarantees the formation of biologically active (2*S*)-flavanones. In our report, docking analysis predicted that *Ct*CHI1 might catalyze 2′,4′,4,6′-tetrahydroxychalcone into naringenin. In vitro experiment validated that (2*S*)-naringenin was generated as an active product and was the metabolic precursor of flavonol glucosides and anthocyanin pigments. This indicated that *Ct*CHI1 was an active enzyme, which belongs to type I CHI protein.

According to lot documents, overexpressing CHI can enrich flavonol content and negatively regulate anthocyanin production, such as petunia and tobacco [30, 31]. In *Arabidopsis*, flavonoid structural enzymes, including CHS, CHI, F3H, and DFR, assemble as a macromolecular complex that interacts in an orientation-dependent manner to ensure efficient flavonoid production [32–34]. In tobacco, overexpression of *Ct*CHI1 raised 4CL expression level and restrain anthocyanidin accumulation in tobacco. More interesting, overexpression of *Ct*CHI1 promoted kaempferol accumulation but inhibited quercetin production, which is different from the previous report [35]. Our results demonstrate that *Ct*CHI1 executes an alteration regulation model in flavonol pathway. The detailed molecular mechanisms of how *Ct*CHI1 increases flavonoids-related secondary metabolites remain to be elucidated. Our metabolomics analysis provides a clue that 788 metabolite compounds were identified as differential markers, and most of them increased significantly (*p* < 0.05) in response to ovx-*Ct*CHI1. Meanwhile, HSYA, rutin, kaempferol-3-*O*-β-rutinoside, and dihydrokaempferol were up-regulated in transgenic safflower lines. The results indicate that *Ct*CHI1 directly interacts with CHS and FLS to promote flavonol and chalcone accumulation, which plays a role as a flavonoid enhancer in safflower.

## Conclusion

The full length of *Ct*CHI1 from safflower floret was an active *Ct*CHI1, which belongs to type I CHIs and located in the nucleus. *Ct*CHI1 plays a role as suppressor in the anthocyanin and quercetin biosynthetic pathway in tobacco. Nevertheless, unlike tobacco, *Ct*CHI1 is a potential enhancer for chalcone and flavonol compounds in safflower. Our study provides a basis to investigate the molecular mechanism of *Ct*CHI1. In the future, further mechanism and more sight remain to be studied in other plants.

## Methods

### Plant materials

Tobacco seeds (*Nicotiana tabacum* L. cv. W38) from BIORUN *Co*. *Ltd* (China) were sterilized with 15% NaClO for 10 min followed by washing three times with sterile distilled water. Seeds were germinated on solidified MS medium (pH 5.8). Tobacco plants were cultivated in a growing room at 25 °C with 16−/8-h light and darkness. The light intensity is set as 22,000 lx. XHH002 line was collected from Chinese Safflower Germplasm Resources in Academy of Agricultural Sciences of Xinjiang. XHH002 safflower line was cultivated in a green-house of Second Military Medical University (SMMU) at 25 °C with the photoperiod of 16-h light and 8-h darkness, and the supplementary light was provided by a high-pressure sodium lamp. It was identified as *Carthamus tinctorius* L. by professor Meili Guo. The Voucher specimen was SMMU141205. They have been deposited in Medicinal Plant Herbarium of Department of Pharmacognosy, School of Pharmacy, Second Military Medical University. Blooming flowers were collected and immediately frozen in liquid nitrogen and stored at − 70 °C. *Nicotiana Benthamiana*, obtained from college of life, FuDan University, was also planted for subcellular localization.

### Amplification of the full-length *Ct*CHI1 cDNA

Total RNA was extracted by TRIzol™ reagent according to the manufacturer’s instructions (Tiangen, China). The 5′ and 3′ cDNA libraries of safflower were constructed by the Clontech Smart™ RACE cDNA amplification kit (Clontech, USA). After primers were designed (***Ct*CHI1**-GSP1: ACACCATGCTCCCCAATCACTGACTCG; ***Ct*CHI1**-GSP2: ATCGTCTTTCCGCCCTCCGTCAAGCC), PCR was carried out using the Advantage®2 PCR Kit (Clontech, USA). The conditions were 5 cycles: 94 °C for 30 s, 72 °C for 3 min; 5 cycles: 94 °C for 10 s, 70 °C for 30 s, 72 °C for 3 min; 30 cycles: 94 °C for 10 s, 68 °C for 30 s,72 °C for 3 min. The PCR products were separated on 1.0% agarose gels (QIAquick® Gel Extraction Kit, Qiagen, Germany) and sequenced. Basing on the sequence assembly of the 5′- and 3′-RACE products, we tried to design full-length primers (forward primer: ***Ct*CHI1**–5′-ACCTGTTTTACTAGTTTCAGGATCG-3′, reverse primer: ***Ct*CHI1**–5′-TTCTCCTAGGCAACTACAATGGC-3′). Subsequently, PCR was performed by using of cDNA template (TransScript® One-step gDNA Removal and cDNA Synthesis SuperMix, TransGen, China) and with high-fidelity KOD-Plus-Neo polymerase (Toyobo, Japan) under the following conditions: 2 min at 94 °C for preheat, 30 cycles of 10 s denaturation at 94 °C, 30 s annealing at 58 °C, and 60 s amplification at 72 °C, hold at 4 °C. The PCR product was cloned into pMD 19 T vector (Takara, Japan). The recombinant plasmids were extracted by using QIAquick® Spin Plasmid Mini-prep kit (Qiagen, Germany) and then sequenced.

### Bioinformatics analysis

BLASTn and BLASTx algorithms were performed in the GenBank database to detect gene annotation. The nucleotide sequences were translated to identify open reading frames (ORFs) by way of the ORF Finder tool in the NCBI database (https://www.ncbi.nlm.nih.gov/.). Multiple-sequence alignment of *Ct*CHI1 and other CHI proteins was performed using DNAMAN. Phylogenetic analysis of *Ct*CHI1 was aligned with that of Clustal W [36] and evolutionary analysis was performed with the NJ method using MEGA4.0 software [37] with 1000 times replication for a bootstrap test. Furthermore, the theoretical isoelectric point (pI) and molecular weight were predicted on the ExPASy server (http://web.expasy.org/compute_pi/) [38–40].

### Subcellular localization

The entire coding sequence (CDS) of *Ct*CHI1 excluding a stop codon was amplified by PCR. The resulting plasmid was constructed as previously described [18]. The construct is confirmed by sequencing and transformed into the *E.* DH5a. The localization of the fusion protein was detected in *N. Benthamiana* cells under a confocal laser-scanning microscopy (Leica TCS SP5, Germany). The bright-field image, GFP fluorescence, and DAPI were shot simultaneously and merged. Green fluorescent protein was detected under the wavelength of 488 nm, whereas DAPI staining was detected under the wavelength of 364 nm. Images were captured with the LAS AF Lit software (Leica, Germany).

### Heterologous expression and purification of *Ct*CHI1 in *E. coli*

To express protein in *E. coli*, *Ct*CHI1 was amplified with seamless cloning primer (forward primer: GGAAGGATTTCACATATGTCCATGGCATCCTTAACCGATAT; reverse primer: ATTTAATTACCTGCAGGGCTTTCAGCGGCAATGGGGGTGG), and the PCR program is the same as cloning the full length of *Ct*CHI1 (Tm depends on the special primer). The PCR product was cloned into the pMAL-c5X vector (after digested by *Nco*I and *Bam*HI). The recombinant plasmid (pMAL-c5X–CHI1) was then transformed into *E. coli* BL21 (DE3) pLys (NEW ENGLAND Biolabs, USA) and sequenced. Transformant cells were grown in 2 ml LB medium containing 100 mg/L ampicillin and 34 mg/L hygromycin with shaking at 37 °C. Then, the culture was incubated in 50 ml fresh LB medium containing ampicillin and hygromycin. It was grown at 37 °C when the optical density of the culture reached approximately 0.6 to 0.8 at 600 nm. 0.5 mM isopropyl 1-β-d-thiogalactoside was added to the medium to induce the protein expression, followed by cultivation at 30 °C for an additional 5 h.

All steps were performed at 4 °C. The resulting cells were harvested by centrifugation at 5000×g for 10 min and resuspended in 1 × phosphate-buffered saline buffer, pH 8.0. The cell suspension was lysed by sonication. The expression protein was purified with an Amylose Resin affinity column (pMAL™ Protein Fusion & Purification System, NEW ENGLAND Biolabs) and analyzed by SDS-PAGE according to a method established by Laemmli [41]. The enzyme solution was concentrated with an Amicon Ultra-15 Centrifugal Filter Device (30,000 MWCO; Millipore, Billerica, MA, USA).

### Enzymatic assay

The reaction mixture (100 μl) consisted of 20 mM 2′,4′,4,6′-tetrahydroxychalcone (dissolved in methanol), 50 mM HEPES-NaOH, pH 7.4 and enzyme. The mixture without enzyme is treated as a control group. The compound was analyzed with an Agilent HPLC series 1100 equipped with a UV detector and a Waters Symmetry C18 column (5 μm particle size, 4.6 mm × 250 mm by C18 reversed-phase). The column was previously equilibrated with 0.2% (v/v) formic acid in water and 100% acetonitrile at 7:3 (v/v). After injection (20 μl), the column was initially developed at a flow rate of 1.0 ml/min at 25 °C and UV detection was monitored at 254 nm.

### Plant transformation

The ORF of *C*tCHI1 was cloned into the binary vector pMT39 to generate the pMT39-*Ct*CHI1 resulting construct subject to the CaMV35S promoter. The detailed steps are the following: the mixture of plasmid and *A. tumefaciens* GV3101 competent cell was kept on ice for 5 min, frozen in liquid nitrogen for 5 min, placed on 37 °C water bathing for 5 min, and finally on ice for 5 min. The resulting construct was transformed into agrobacterium GV3101. The infiltration medium for resuspension of the bacteria contained 0.02% (v/v) Silwet L-77 and 5% sucrose (w/v). Plant transformation was performed as described elsewhere with the *Agrobacterium*-mediated leaf disk transformation [42]. Transformed plants were identified by genomic PCR. Five representative transformants were screened out for the following analysis. In the meantime, the negative control (CK) was set with empty-vector. The *Agrobacterium* GV3101 resuspension with resulting construct was transformed into safflower through a pollen tube as previously described by our team [18].

### Quantitative RT-PCR analysis

Total RNA (1 μg) was reverse transcribed using the first-strand cDNA synthesis kit (TransGen Biotech, Beijing, China). The real-time PCR was performed by the instructions of the SYBR Green Real-time Master Mix kit (Toyobo, Japan) and carried out in ABI 7500 system (ABI, USA). The PCR condition and specific primers were cited [18, 43]. The housekeeping gene 60S (60S acidic ribosomal protein) was normalized as reference gene. The fold change of genes was calculated by way of the 2^-ΔΔCt^ method. Standard deviations were combined from three independent replicates.

### Western blotting analysis

Total protein was extracted by protein isolation buffer, including 45 ml 1 M Tris-HCL (pH 8.0), 75 ml glycerol, 6 g polyvinylpyrrolidone, adding sterile water to 300 ml. Western blot was carried out with the primary antibody of EGFP (Enhanced Green Fluorescent Protein) from mouse and secondary antibody of IgHRP from goat. Actin protein was treated as reference protein. Tanon 5200 (China) was used to capture the images automatically.

### Metabolite analysis in transgenic tobacco and safflower

Frozen flower samples at blooming stage in tobacco were powdered and weighed right away. Three representative compounds (kaempferol, quercetin, and anthocyanins) in tobacco were analyzed using HPLC. Experiments on extraction of the three compounds were performed as described previously with the following modification [34]. In short, kaempferol and quercetin were extracted from approximately 200 mg of finely ground tobacco flowers in 2 ml 1% HCl/methanol (v/v), simultaneously, anthocyanin was isolated from ground flower dissolved by 80% methanol. Kaempferol and quercetin extractions were first sonicated for 30 min, and then held at 4 °C overnight, whereas anthocyanin extraction was only held at 4 °C for 24 h. In this experiment, all representative compounds were determined as aglycones by way of acid-hydrolyzed extraction. After centrifuge treatment, 400 μl of the flavonol supernatant was shifted to a new tube, acid-hydrolyzed by adding 120 μl of 3 N HCl, incubated for 1 h at water-bath 90 °C, and then mixed with 200 μl of methanol. On the other hand, 200 μl of the anthocyanins supernatant was shifted to a new tube, acid hydrolyzed by adding 220 μl of 3 N HCl, incubated up to 3 h at water-bath 90 °C and then mixed with 200 μl of methanol. Prior to uploading the samples, the hydrolyzation solution was supposed to be carefully filtered through a 0.22 μm filter membrane (Millipore, USA). An Agilent1100 series HPLC system equipped with an Agilent TC-C_18_ column (5 μm, 4.6 × 250 mm) was used for chromatographic analysis. Flow rate was 0.4 ml/min. The wavelength of kaempferol and quercetin was 350 nm, whereas anthocyanin was detected at 650 nm. The three standard compounds were purchased from Sigma-Aldrich (St. Louis, MO, USA) and Yuanye Bio Int (China). The standard curve is drawn and calculated with R^2^ (approximately 0.997–0.999). Sample preparation for secondary metabolite analysis in transgenic safflower was described as previously mentioned [18]. Blooming flowers were collected and placed in liquid nitrogen right away. Frozen flower samples were dried at 50 °C and ground to fine powder for HLPC analysis.

### Chromatography and mass spectrometry conditions for metabolomics in transgenic safflower

HPLC-time-of-flight analysis was completed with an Agilent 1100 LC system equipped with an Agilent 6220 mass spectrometer (Agilent, USA). Chromatographic separation was performed on a Waters ACQUITY UPLC BEH C_18_ column (100 mm × 2.1 mm, 2.5 μm, Waters Corporation, USA). The mobile phases were made up of solution A (0.1% aqueous formic acid) and solution B (acetonitrile with 0.1% formic acid). The applied elution conditions were as follows: 0 to 2 min, 5% B; 2 to 2.5 min, 5 to 15% B; 2.5 to 7.5 min, 15% B; 7.5 to 8 min, 15 to 20% B; 8 to 10 min, 20 to 21% B; 10 to 18 min, 21–95% B; 18 to 25 min, 95% B. Post-time was 10 min. The injection volume was 5 μl, and the flow rate was set at 0.50 ml/min. The temperatures of the autosampler and analytical chromatographic column were set at 4 °C and 25 °C, respectively. An electrospray ionization source was carried out in negative modes. The negative mode conditions were as follows: capillary voltage, 3.5 kV; drying gas flow, 11 L/min; gas temperature, 350 °C; nebulizer pressure, 45 psi; frag mentor voltage, 165 V; skimmer voltage, 65 V. The mass spectrum was set from 100 to 1200 *m/z* in centroid mode. Samples were overviewed by TIC representation. The HPLC-MS data were exported for PCA and PLS-DA (VIP > 1.0, *p* < 0.05).

## Additional files

Additional file 1: **Figure S1.** (A). Standard curve of chalconenaringenin. (B). Standard curve of naringenin (C). Consumption of chalconenaringenin and production of naringenin during catalytic reaction for first 20 min. (TIF 2080 kb)

Additional file 2: **Figure S2.** (A) Relative transcription abundancy of flavonoid-related genes in individual transgenic tobacco line. (B) Flavonoids contents of fresh weights (μg/mg) in individual transgenic tobacco line. Error bar is mean ± SD, data represent biological duplication. * *p* ≤ 0.05, ** *p* ≤ 0.01. (TIF 5762 kb)

Additional file 3: **Figure S3.** (A) Relative transcription abundancy of flavonoid-related genes in individual transgenic safflower line. (B) Relative content changes of compounds implicating in flavonoids pathway in individual safflower line. Error bar is mean ± SD, data represent biological duplication. * *p* ≤ 0.05, ** *p* ≤ 0.01. (TIF 190 kb)

Additional file 4: **Table S1.** Differential metabolite ions from the negative ion mode were summarized. (XLSX 51 kb)

## Data Availability

All data supporting the findings is contained in the manuscript and its supplementary files.

## Abbreviations

ANS: anthocyanidin synthase
bHLH: Basic helix-loop-helix
CDS: Coding sequence
CHI: Chalcone isomerase
CHS: Chalcone synthase
CK: Negative control
DFR: dihydroflavonol 4–reductase
F3H: Flavanone 3-hydroxylase
HPLC: High Performance Liquid Chromatography
HSYA: hydroxysafflor yellow A
IPTG: Isopropyl 1-β-D-thiogalactoside
MW: Molecular weight
MYB: myeloblastosis
ORF: Open reading frames
PCA: Principal component analysis
pI: Theoretical isoelectric point
PLS-DA: Partial least squares discriminant analysis
qPCR: Quantitative real-time PCR
TIC: Total ion chromatogram
