# Supplementary figures and images for: Integrating molecular characterization and metabolites profile revealed CtCHI1’s significant role in Carthamus tinctorius L
Source: BMC Plant Biol. 2019 Aug 27;19:376. doi: 10.1186/s12870-019-1962-0 (PMC6712624; doi:10.1186/s12870-019-1962-0)

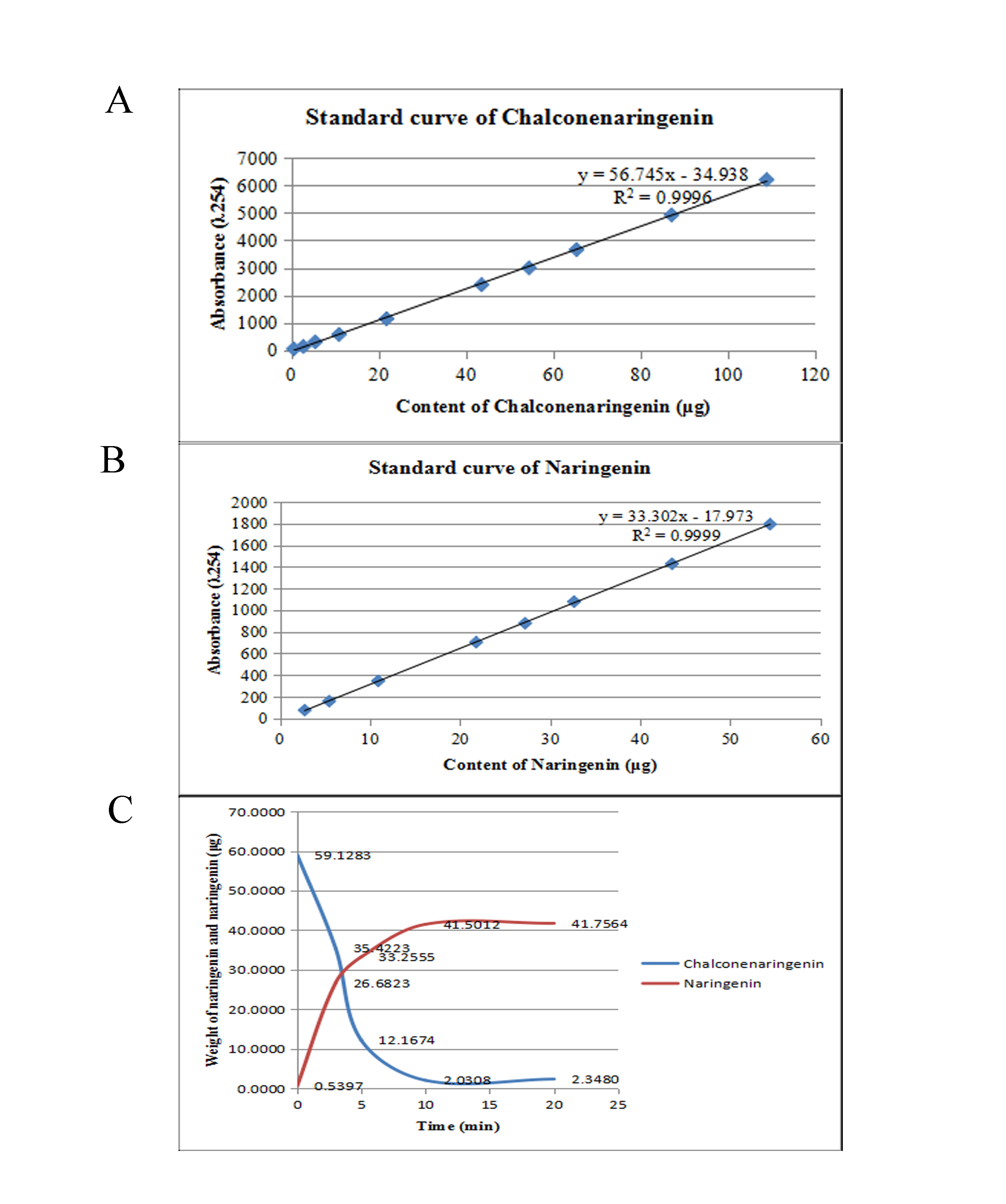

Supplement: Supplementary file 1 — Figure S1. (A). Standard curve of chalconenaringenin. (B). Standard curve of naringenin (C). Consumption of chalconenaringenin and production of naringenin during catalytic reaction for first 20 min. (TIF 2080 kb) [file 12870_2019_1962_MOESM1_ESM.tif]

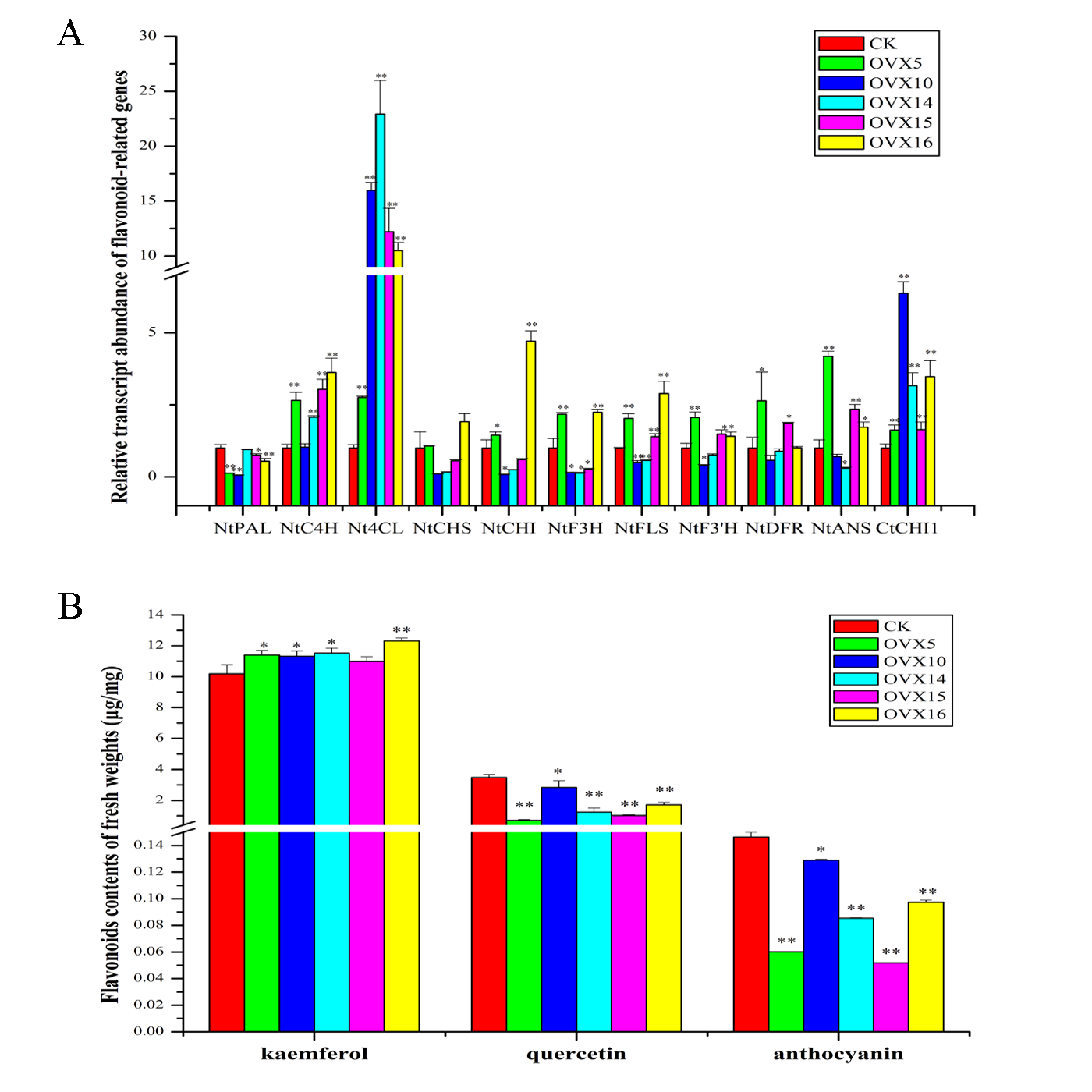

Supplement: Supplementary file 2 — Figure S2. (A) Relative transcription abundancy of flavonoid-related genes in individual transgenic tobacco line. (B) Flavonoids contents of fresh weights (μg/mg) in individual transgenic tobacco line. Error bar is mean ± SD, data represent biological duplication. * p ≤ 0.05, ** p ≤ 0.01. (TIF 5762 kb) [file 12870_2019_1962_MOESM2_ESM.tif]

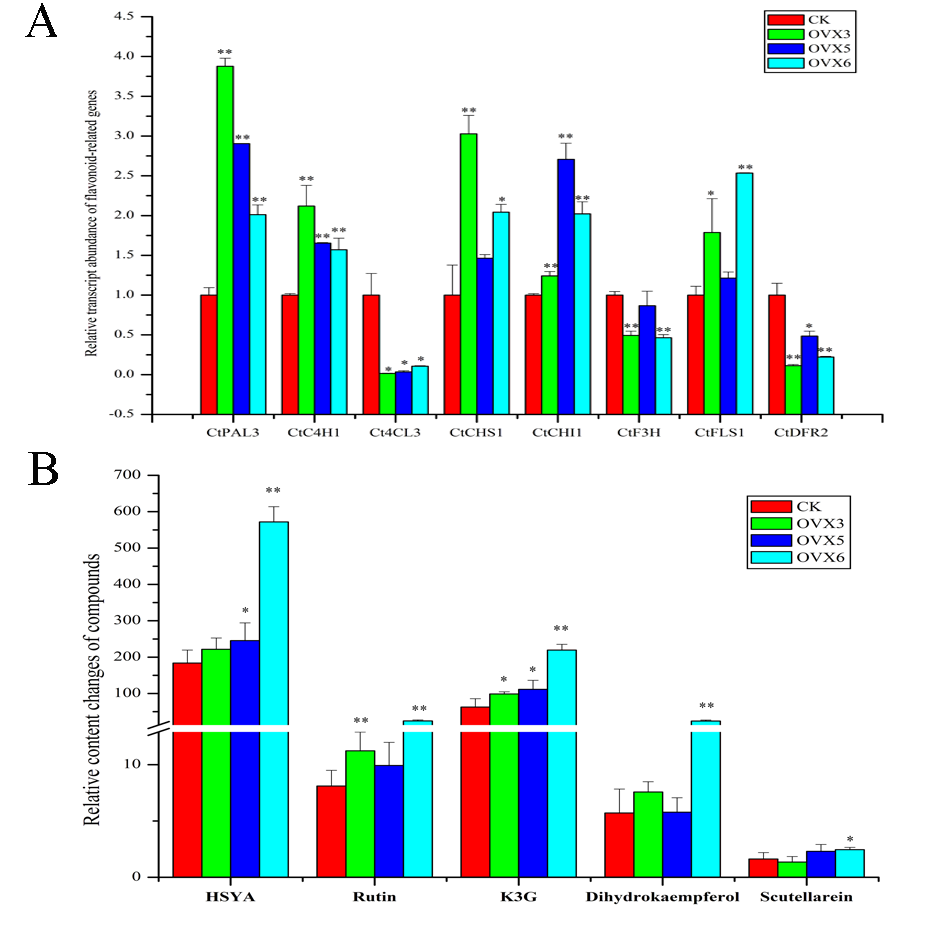

Supplement: Supplementary file 3 — Figure S3. (A) Relative transcription abundancy of flavonoid-related genes in individual transgenic safflower line. (B) Relative content changes of compounds implicating in flavonoids pathway in individual safflower line. Error bar is mean ± SD, data represent biological duplication. * p ≤ 0.05, ** p ≤ 0.01. (TIF 190 kb) [file 12870_2019_1962_MOESM3_ESM.tif]
